# Early Expulsion of the Placenta

**Published:** 1892-05

**Authors:** F. C. Ferguson


### Selections and Abstracts.

### EARLY EXPULSION OF THE PLACENTA.

If there be danger to the child in forcing an early expulsion of the placenta, the danger to the mother is equally as great, and probably greater. The practice is almost certain to cause a retention of portions of the secundines, which worry and fret the uterus, causing prolonged post-partum pains, frequently post-partum hemorrhage, and various other dangerous puerperal accidents, such as metritis, peritonitis, septicæmia, etc.

The conclusion of the whole matter is, that unless there be some positive indications for interference, such as post-partum hemorrhage, the physician should wait until spontaneous retraction of the uterus has expelled the placenta into the vagina before making attempts to deliver it. It may then be extracted without fear of injurious consequences.

When physicians generally learn this valuable lesson, post-partum complications, tardy puerperal convalescence, and cases of chronic invalidism, resulting from mismanagement of the third stage of labor, will be much rarer than at the present time.—*F. C. Ferguson, in Indiana Medical Journal.* —*Nash. Journal of Med. and Surg., Apr. 1892, p. 176.*

